# Study of the relationship between cognitive deficits and sociodemographic, clinical and therapeutic factors in patients with schizophrenia

**DOI:** 10.1192/j.eurpsy.2025.2268

**Published:** 2025-08-26

**Authors:** N. Smaoui, D. Jardak, N. Charfi, I. Gassara, R. Feki, M. Bou Ali Maalej, J. Ben Thabet, M. Maalej, S. Omri, L. Zouari, L. Triki

**Affiliations:** 1 Psychiatry C, Hedi Chaker university hospital; 2 Functional Explorations, Habib Bourguiba University Hospital, Sfax, Tunisia

## Abstract

**Introduction:**

Cognitive deficits are a fundamental characteristic of schizophrenia, similar to positive and negative symptoms. They lead to significant disability due to their impact on various domains of life.

**Objectives:**

This study aimed to study the relationship between cognitive impairment and sociodemographic, clinical, and therapeutic factors in patients with schizophrenia.

**Methods:**

This study was carried out in the Psychiatry « c » Department at Hedi Chaker University Hospital in Sfax, Tunisia, involving 15 patients with schizophrenia. We used the Screen For Cognitive Impairment in Psychiatry (SCIP) in its literary Arabic version to assess cognitive functions. Data were analyzed using SPSS version 20.0 software.

**Results:**

The mean age of the patients was 40 ± 12.72 years. Among the participants, 80% (n=12) were single. Seven cases (46.7%) had not exceeded primary education. The mean age of illness onset was 27.8 years, and the mean duration of illness was 13.7 years. Five patients (33.3%) had a family history of psychiatric disorders. All patients were receiving antipsychotics (AP), and 13.2% of them were on Haloperidol decanoate (HD). The mean scores for the total SCIP (ST) and its five subscales (verbal learning test-immediate (VLT-I), working memory test (WMT), verbal fluency test (VFT), verbal learning test-delayed (VLT-D), and processing speed test (PST)) were 37.40, 12.87, 14.27, 3.93, 2.47, and 3.93, respectively. A negative correlation was found between age and performance on the ST, WMT, and PST (r values: -0.515, -0.629, -0.615, respectively). Regarding marital status, VLT-I scores were better in single patients (p=0.007). Our study revealed that the low level of education was significantly correlated with several cognitive tests measured by the SCIP, including the ST, VLT-I, WMT, VFT, and PST. Mean scores for ST, VLT-I, WMT, VFT and PST were significantly lower in patients with illness onset after age 40 (p<0.05). The WMT score was significantly lower in patients with an illness duration exceeding 5 years and in patients with a family history of psychiatric disorders (p values: 0.05; 0.021). The PST score was significantly lower in patients on HD (p=0.038).

**Conclusions:**

Sociodemographic, clinical, and therapeutic factors harm the cognitive abilities of patients with schizophrenia. Thus, it is essential to carry out neurocognitive assessments during the follow-up of these patients, taking into account factors likely to predict cognitive impairment.

**Disclosure of Interest:**

None Declared

